# Consuming Mushrooms When Adopting a Healthy Mediterranean-Style Dietary Pattern Does Not Influence Short-Term Changes of Most Cardiometabolic Disease Risk Factors in Healthy Middle-Aged and Older Adults

**DOI:** 10.1016/j.tjnut.2023.12.026

**Published:** 2023-12-20

**Authors:** Cassi N Uffelman, Jacqueline N Schmok, Robyn E Campbell, Austin S Hartman, Matthew R Olson, Nicole L Anderson, Nichole A Reisdorph, Minghua Tang, Nancy F Krebs, Wayne W Campbell

**Affiliations:** 1 Department of Nutrition Science, Purdue University, West Lafayette, Indiana, United States; 2 Department of Statistics, Purdue University, West Lafayette, Indiana , United States; 3 Department of Biological Science, Purdue University, West Lafayette, Indiana , United States; 4 Skaggs School of Pharmacy and Pharmaceutical Sciences, University of Colorado Anschutz Medical Campus, Aurora, Colorado, United States; 5 School of Medicine, Department of Pediatrics, University of Colorado Anschutz Medical Campus, Aurora, Colorado, United States

**Keywords:** fungi, cardiovascular diseases, type 2 diabetes, *Agaricus bisporus*, *Pleurotus ostreatus*

## Abstract

**Background:**

Mushrooms are a nutritious food, though knowledge of the effects of mushroom consumption on cardiometabolic risk factors is limited and inconsistent.

**Objective:**

We assessed the effects of consuming mushrooms as part of a healthy United States Mediterranean-style dietary pattern (MED) on traditional and emerging cardiometabolic disease (CMD) risk factors. We hypothesized that adopting a MED diet with mushrooms would lead to greater improvements in multiple CMD risk factors.

**Methods:**

Using a randomized, parallel study design, 60 adults (36 females, 24 males; aged 46 ± 12 y; body mass index 28.3 ± 2.84 kg/m^2^, mean ± standard deviation) without diagnosed CMD morbidities consumed a MED diet (all foods provided) without (control with breadcrumbs) or with 84 g/d of *Agaricus bisporus* (White Button, 4 d/wk) and *Pleurotus ostreatus* (Oyster, 3 d/wk) mushrooms for 8 wk. Fasting baseline and postintervention outcome measurements were traditional CMD risk factors, including blood pressure and fasting serum lipids, lipoproteins, glucose, and insulin. Exploratory CMD-related outcomes included lipoprotein particle sizes and indexes of inflammation.

**Results:**

Adopting the MED-mushroom diet compared with the MED-control diet without mushrooms improved fasting serum glucose (change from baseline −2.9 ± 1.18 compared with 0.6 ± 1.10 mg/dL; time × group *P* = 0.034). Adopting the MED diet, independent of mushroom consumption, reduced serum total cholesterol (−10.2 ± 3.77 mg/dL; time *P* = 0.0001). Concomitantly, there was a reduction in high-density lipoprotein (HDL) cholesterol, buoyant HDL2b, and apolipoprotein A1, and an increase in lipoprotein(a) concentrations (main effect of time *P* < 0.05 for all). There were no changes in other measured CMD risk factors.

**Conclusions:**

Consuming a Mediterranean-style healthy dietary pattern with 1 serving/d of whole *Agaricus bisporus* and *Pleurotus ostreatus* mushrooms improved fasting serum glucose but did not influence other established or emerging CMD risk factors among middle-aged and older adults classified as overweight or obese but with clinically normal cardiometabolic health.

**Trial registration number:**

https://www.clinicaltrials.gov/study/NCT04259229?term=NCT04259229&rank=1

## Introduction

Mushrooms, edible fungi, have a unique nutritional profile that may be underappreciated as a functional food for health. Worldwide, mushrooms have been consumed for thousands of years for nutritional and medicinal purposes; currently, the average consumption is ∼5 kg per person per year [1]. In contrast, mushroom consumption among Americans is considerably lower. The average intake of the most consumed species, *Agaricus bisporus* (white button, crimini, portabella), is <1.4 kg per person per year [2], equating to ∼1.5 medium mushrooms per week. From a whole food perspective, mushrooms have a nutritional profile consistent with the recommendations set forth by the Dietary Guidelines for Americans (DGA) including low in energy and cholesterol, fat-free, and very low in sodium [3,4].

From a nutrient perspective, mushrooms contain several vitamins and minerals including riboflavin, niacin, pantothenic acid, selenium, copper, and potassium, making them a healthful food choice [4,5]. They have multiple bioactive compounds that exhibit cardioprotective properties, primarily demonstrated in cell and animal models [[6], [7], [8]]. Namely, mushrooms are the primary dietary source of the amino acid, L-ergothioneine, which is not synthesized by animals or higher plants [9]. L-ergothioneine is associated with protection from several chronic diseases, including cardiometabolic diseases (CMDs), and appears to be important for healthy aging through its antioxidant and anti-inflammatory properties [[10], [11], [12]]. Notably, the concentration of bioactive compounds, including L-ergothioneine, differs among mushroom varieties. Although *Pleurotus ostreatus* (gray oyster) mushrooms are among the highest containing sources of L-ergothioneine (∼14 mg/100 g) [5], *Agaricus bisporus* (white button, crimini, portabella) contains much lower amounts ranging from 1 to 4 mg/100 g [4,13,14]. Other bioactive compounds including polysaccharides, such as β-glucans, have roles in immune modulation, and glucose and lipid control [15]. Fungal lectins, terpenoids, alkaloids, and statins (for example, lovastatin) have potential immunomodulatory, anti-inflammatory/antioxidant, neuroprotective, and cholesterol-lowering properties, respectively [[16], [17], [18], [19]].

Despite the presence of several health-promoting compounds in mushrooms, a paucity of research has assessed the effects of mushroom consumption on indices of cardiometabolic health in humans [[20], [21], [22], [23]]. In our 2023 systematic review, we found evidence from experimental research consistently supports a positive effect of mushroom consumption (of all species) on fasting serum/plasma triglycerides and high-sensitivity C-reactive protein (hs-CRP), but not on other cardiometabolic health outcomes (other lipids/lipoproteins, measures of glucose control, or blood pressures) [20]. Other systematic reviews have reported favorable impacts of several mushroom species, including *Agaricus bisporus* and *Pleurotus ostreatus*, on glucose control (fasting and/or postprandial glucose), lipids and lipoproteins (triglycerides, LDL- and/or total cholesterol), and/or markers of inflammation (TNF-α, adiponectin, and oxygen radical absorbance capacity) [[21], [22], [23]]. Although limited evidence suggests multiple potential health benefits of mushroom consumption, limitations of the literature (for example, study methodology including lack of dietary control and poor reporting) warrant more in-depth research to validate these findings.

This research aims to assess the effects of including mushrooms as part of a healthy United States Mediterranean-style dietary pattern (MED) on traditional and emerging risk factors for CMDs. We hypothesize that consuming mushrooms as part of a MED diet will lead to greater improvements in multiple traditional risk factors for CMDs compared with MED without mushrooms. In addition, this study measured other emerging CMD risk outcomes (that is, lipoprotein particle sizes and inflammation indexes) in an exploratory manner because of the paucity of human research addressing these important topics.

## Methods

### Experimental design

Using a randomized, parallel study design, healthy middle-aged and older adults (*n* = 60, 30/group) completed a 10-wk trial including a 2-wk baseline period followed by an 8-wk dietary intervention. During the 8-wk intervention, participants consumed a fully controlled, euenergetic, weight-maintenance MED diet with or without (control) mushrooms. Outcome measurements including traditional and emerging risk factors for CMDs were assessed during a standardized test day at baseline and postintervention. Traditional risk factors for CMDs included systolic and diastolic blood pressures, and fasting serum lipids/lipoproteins (total, HDL, LDL, and non-HDL cholesterol, triglycerides), glucose, and insulin. Exploratory outcomes were emerging risk factors for CMDs including lipoprotein particle sizes and blood markers of inflammation. The study protocol was approved by the Purdue University Institutional Review Board (IRB 2019-650) and was registered in the public trial registry Clinicaltrials.gov (NCT04259229) before participant recruitment commenced. All participants provided written consent and received monetary compensation for their time.

### Eligibility criteria

Participants were recruited from Lafayette, IN, in the United States, and were male or female, age 30–69 y, with overweight or class 1 obesity classification (BMI 25.0–34.9 kg/m^2^). Additional inclusion criteria were as follows: not severely or extremely depressed (Beck’s Depression Inventory score ≤ 30); fasting serum total cholesterol <240 mg/dL; LDL cholesterol <160 mg/dL; triglycerides <400 mg/dL; glucose <110 mg/dL; systolic/diastolic blood pressure <140/90 mm Hg; body weight stable for 3 mo before enrollment (± 3 kg); stable physical activity regimen 3 mo prior; medication use stable for 6 mo prior; nonsmoking; nondiabetic; not acutely ill; females not pregnant or lactating. Participants were required to consume the prescribed diets and travel to testing facilities.

Upon study qualification, participants were randomly assigned to either the control group (MED-control) or the mushroom group (MED with mushrooms), using an online randomization plan generator (http://randomization.com, seed 7433).

### Dietary intervention and baseline dietary assessment

During the 2-wk baseline period, participants consumed their habitual, self-selected diets.

Dietary intake data for 24-h recalls were collected on 3 nonconsecutive days and included ≥1 weekend day. Data were analyzed using the Automated Self-Administered 24-h Dietary Assessment Tool, version (2020), developed by the National Cancer Institute, Bethesda, MD [24]. Dietary intake data were used to calculate the total Healthy Eating Index (HEI-2015) score as previously described [25], which quantifies how well individuals’ dietary intakes align with the Dietary Guidelines for Americans (DGA) recommendations.

During the 8-wk intervention period, all participants consumed a controlled, euenergetic, weight-maintenance MED diet with or without (control) mushrooms. A Registered Dietitian developed 3 7-d rotating menus corresponding to 3 different energy levels, 2000, 2400, or 2800 kcal/d, using ProNutra software (Viocare, Inc.). Food levels were cross-checked with the recommended daily or weekly food/subgroup intakes to ensure adherence to the MED (Supplemental Table 1). Participant energy requirements were estimated using sex-specific equations developed by the Institute of Medicine [26]. Participants were provided with all the menu-specific foods and beverages throughout the 8-wk controlled feeding period. Most intervention foods were provided to participants using a grocery curbside pick-up service. Select foods, including mushrooms or control “powder,” were provided to participants at Purdue University. Participants in the mushroom group were provided with fresh mushrooms weekly and instructed to consume them 7 d/wk with 84 g/d *Agaricus bisporus* (white button) on 4 d/wk and 84 g/d *Pleurotus ostreatus* (oyster) on 3 d/wk. Participants self-selected which days they consumed each variety and were permitted to consume their mushrooms raw, sauteed (5 min), or microwaved (30 s). Participants in the control group were provided with a weekly container of study “powder” (breadcrumbs) and asked to consume 1 tsp/d, mixed into any meal of their choice. Participants in the control group were provided with study “powder” to ensure equal treatment of groups but remained blind to the substance of the “powder” even after study completion. Participants were not explicitly told which group they were randomly assigned into, but given the nature of the dietary intervention, those in the mushroom group were aware of their assignment.

During the baseline test day, participants were provided with in-person nutrition counseling and given written food storage and preparation instructions in the form of a menu booklet. Participants were requested to fill out the menu booklet daily and return it at the end of each week. Dietary adherence was assessed using data from the weekly reports on the consumption of protocol foods, substitutions, and/or additions of nonprotocol foods to the diet. To promote dietary adherence, participants had frequent communication with the study coordinator, including attending a weekly weigh-in and food (study “powder” or mushroom) pick-up appointment and weekly online or phone conversations.

During the study intervention, participants were asked to discontinue intake of any dietary supplements, maintain their current level of physical activity, and alert the study coordinator of any changes in their health, including medication changes.

### Clinical assessments

During the baseline and week 8 in-clinic testing days, participants reported to the Purdue University clinical research center following a 10-h overnight fast. Upon arrival in a fasting state, participants rested for 15 min in a quiet, dimly lit room, and blood pressures were recorded. A minimum of 2 measurements were recorded and a third was taken if either systolic or diastolic measures were >3 mm Hg different between the first 2 measurements. The multiple systolic and diastolic measurements were each averaged. Next, participants provided a blood sample from the antecubital vein, described in detail below. Bodyweight measurements were obtained at both clinical visits.

### Blood processing and analysis

Blood from the antecubital vein was placed into vacutainers containing a clot activator or EDTA to obtain serum or plasma, respectively. Serum vacutainers were held at room temperature for ≥15 min or until clotting occurred whereas EDTA vacutainers were immediately refrigerated until centrifugation at 4000 × *g* at 4°C for 15 min. Serum samples were shipped to Mid America Clinical Laboratories for a comprehensive metabolic panel and to SpectraCell Laboratories for a lipoprotein particle plus panel. This panel included 5 markers of inflammation: hs-CRP, lipoprotein(a), apolipoprotein B, apolipoprotein A1, and homocysteine. Plasma aliquots were shipped to Heartland Assays for L-ergothioneine analysis using liquid chromatography-tandem mass spectrometry.

### Calculations

The homeostatic model assessment for insulin resistance (HOMA-IR) was calculated as [(fasting glucose mg/dL × fasting insulin μIU/mL)/405] [27].

### Statistical analysis

All data were double entered independently and cross-checked for accuracy by the study coordinator. Our primary analysis follows an intent-to-treat plan analyzed via a 2 × 2 repeated-measures ANOVA using the PROC MIXED command in SAS version 9.4 (SAS Institute) by a statistician who was blinded to the participant group assignment. A linear mixed model was used to *1*) compare MED-control and MED-mushroom preintervention values; *2*) assess the interaction of intervention and time (MED-mushroom changes compared with MED-control changes); *3*) assess the main effect of time if a group × time interaction was not observed (data from both groups pooled). All outcomes of interest were controlled for age, sex, and BMI at each time point. The PROC MIXED command in SAS uses maximum likelihood to account for missing data [28].

A secondary linear model using the PROC GLM command was used to predict postintervention values and differences between groups at postintervention (Supplemental Table 2). Covariates in this model included age, sex, BMI, and baseline values. When confronted with missing data, the second model used regression imputation to fill in missing values on the basis of participant baseline characteristics. Results are presented as adjusted least square (LS) means ± standard error of the LS mean unless otherwise noted. Significance was set at *P* < 0.05 for all outcomes.

Because of the exploratory nature of this research, this study was not designed with power calculations. Instead, *n* = 30 participants/group was selected consistent with that requirement for the consideration of use by the 2015 Dietary Guidelines Advisory Committee in creating future DGA [29].

Raw, unadjusted means, SD, and sample size for each outcome by time point and group are available in Supplemental Table 3. An assessment of the effect size for participants who completed the intervention was calculated using Cohen’s d [30] and is available in Supplemental Table 3.

## Results

### Participants

**FIGURE 1:**
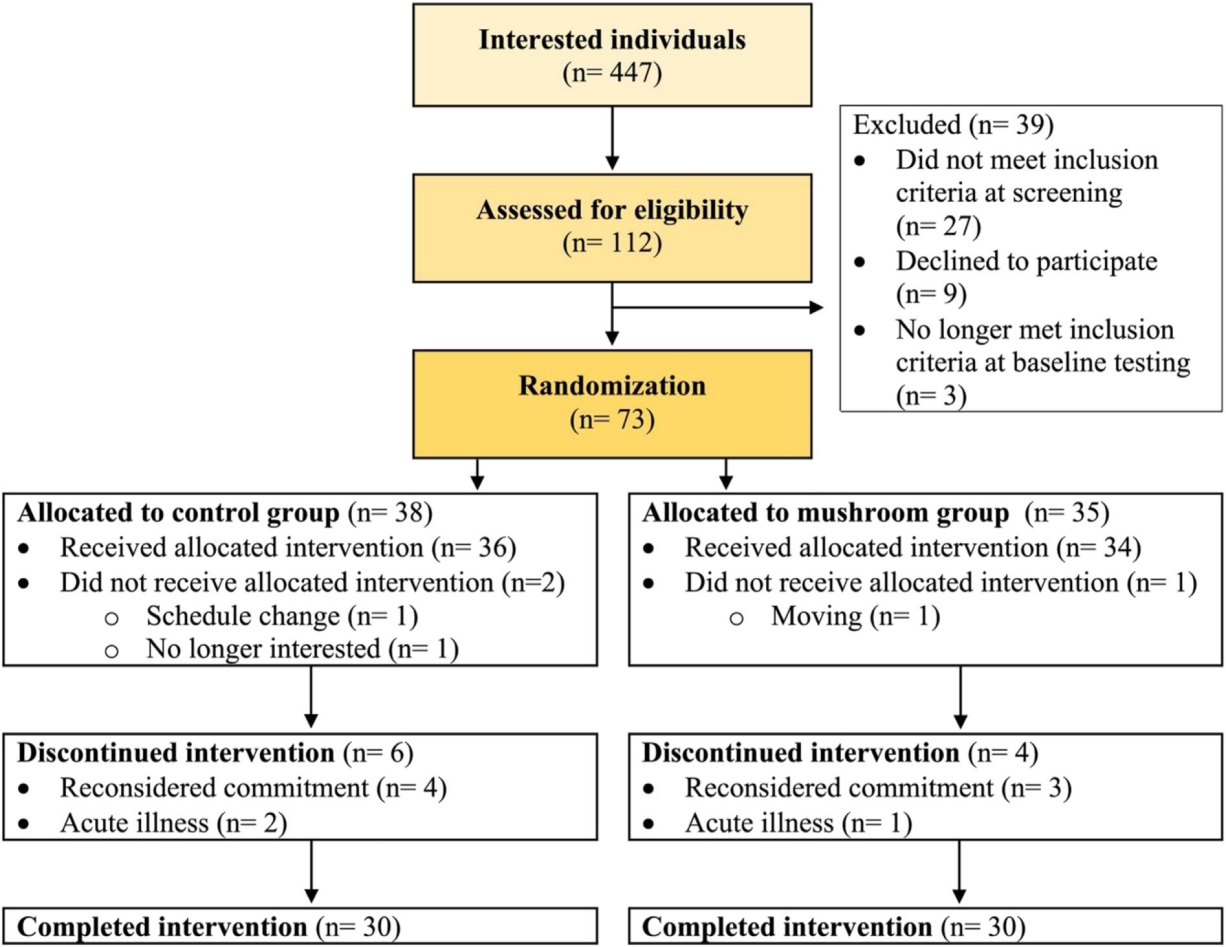
CONSORT participant flow diagram.

During the clinical testing phase (January 2020 to November 2022), the study coordinator was in contact with 447 interested individuals, and 112 individuals were screened for eligibility. Of the 76 participants who consented at the start of the baseline testing day, 73 completed all baseline testing and were randomly assigned to a treatment group. Three participants no longer met the inclusion criteria during the baseline test day (high blood pressure: *n* = 2, not willing to eat the prescribed diet: *n* = 1), and upon realization, the test day was terminated. Three participants who completed baseline testing did not begin the intervention. Of the remaining 70 participants, 10 (control group *n* = 6, mushroom group *n* = 4) dropped out of the intervention, resulting in 60 participants (*n* = 30/group) completing the 8-wk dietary intervention, as detailed in Figure 1. Baseline demographics and fasting clinical characteristics are reported in Table 1. There was no statistically significant difference between groups at baseline for each parameter, except dense LDL IV (*P* = 0.019).

**TABLE 1:** Demographic and fasting clinical characteristics of participants at baseline testing

Demographic characteristics	MED-Control (*n* = 38)	MED-Mushroom (*n* = 34)	Total (*n* = 72)^1^
Age at randomization (y)	44 ± 11	46 ± 12	45 ± 12
Female, *n* (%)	25 (66)	18 (53)	43 (60)
White, *n* (%)	29 (76)	22 (65)	51 (71)
Hispanic or Latin, *n* (%)	6 (16)	5 (15)	11 (15)
Asian, *n* (%)	2 (5)	5 (15)	7 (10)
Black, *n* (%)	1 (3)	1 (3)	2 (3)
Other (not specified), *n* (%)	0 (0)	1 (3)	1 (1)
Weight (kg)	82.4 ± 14.5	85.3 ± 13.9	83.8 ± 14.3
BMI (kg/m^2^)	28.3 ± 2.7	28.5 ± 2.8	28.4 ± 2.8
Fasted clinical characteristics			
Systolic blood pressure (mm Hg)	115 ± 10.2	118 ± 13.1	116 ± 11.7
Diastolic blood pressure (mm Hg)	77 ± 6.7	79 ± 11.2	78 ± 9.0
Total cholesterol (mg/dL)	199 ± 36.3	186 ± 39.8	193 ± 38.3
HDL cholesterol (mg/dL)	57 ± 20.4	52 ± 14.1	55 ± 17.8
LDL cholesterol (mg/dL)	123 ± 29.5	113 ± 32.1	118 ± 31.0
Triglycerides (mg/dL)	103 ± 57.2	123 ± 61.7	113 ± 59.9
Glucose (mg/dL)	91 ± 8.6	94 ± 8.3	92 ± 8.5
BUN (mg/dL)	13 ± 4.1	14 ± 3.2	13 ± 3.7
Creatinine (mg/dL)	0.84 ± 0.15	0.83 ± 0.10	0.84 ± 0.13
eGFR (mL/min/1.73 m^2^)	95 ± 14.5	96 ± 13.9	96 ± 14.1
ALT (U/L)	21 ± 11.6	20 ± 7.9	21 ± 10.0
AST (U/L)	23 ± 8.4	22 ± 6.1	22 ± 7.4

Data are means ± SD. There were no statistically significant differences between groups at baseline.

Abbreviations: ALT, alanine transaminase; AST, aspartate transferase; BUN, blood urea nitrogen; eGFR, estimated glomerular filtration rate.

1 One participant who completed baseline testing and dropped out of the intervention was found to have a baseline AST value classified as an extreme outlier. This participant was removed from the analysis resulting in *n* = 72 at baseline.

### Baseline dietary assessment and adherence to the dietary intervention

The mean HEI-2015 score among the participants who completed baseline 24-h recalls (*n* = 69) was 55 ± 12.0 (mean ± SD) and 57 ± 14.0 for the MED-control and MED-mushroom groups, respectively. A total HEI-2015 score was also calculated for each of the intervention menus and averaged 84 ± 0.38 where the maximum HEI score possible is 100 (Supplemental Figure 1). The HEI score of the MED-mushroom diet was only ∼0.15 arbitrary units greater than the MED-control diet.

Data from weekly menu booklets were used to calculate dietary adherence to the intervention. The average adherence to consuming protocol foods across the 8-wk intervention was 92%. Other indices of adherence included an average of 5 ± 9 nonprotocol food additions (mean ± SD) and 9 ± 11 protocol-acceptable substitutions across the 8-wk intervention. Other blood markers including the changes in blood urea nitrogen (BUN) and L-ergothioneine support adherence to the dietary intervention and consumption of mushrooms, respectively. We observed an increase in BUN (change estimate 2.2 ± 0.45 mg/dL; time effect *P* < 0.0001), consistent with adherence to a higher protein diet relative to habitual intake (21% compared with 18% of total energy intake from protein). Plasma L-ergothioneine increased in the mushroom group by 3.64 ± 0.26 μM and was not changed in the control group (change estimate −0.10 ± 0.25; time × group interaction *P* < 0.0001; Supplemental Table 4), consistent with participants consuming the mushrooms as advised.

### Traditional CMD risk factors

Adoption of the MED diet with mushrooms improved (reduced) fasting glucose (time × group *P* = 0.034). Postintervention, fasting glucose trended lower (2.1 mg/dL difference; *P* = 0.082) for the mushroom compared with control group. The evaluation of the effect size using Cohen’s d indicates a small effect (−0.49) of the MED diet with mushrooms on changes in fasting glucose. The adoption of a MED diet, independent of mushroom intake, reduced total and HDL cholesterol (main effect of time *P* < 0.05). There were no changes in systolic or diastolic blood pressures, other blood lipids or lipoproteins (LDL cholesterol, non-HDL cholesterol, triglycerides), insulin, or HOMA-IR with the adoption of either MED diet (Table 2).

**TABLE 2:** Effects of consuming a MED diet with or without mushrooms for 8 wk on traditional risk factors for cardiometabolic diseases

Outcome	MED-Control			MED-Mushroom			*P* values	
	Baseline	Post	Change	Baseline	Post	Change	Time	Time × Group
Systolic blood pressure (mm Hg)	116 ± 1.9	114 ± 2.0	−1.8 ± 1.56	118 ± 1.9	116 ± 2.0	−2.3 ± 1.60	0.075	0.808
Diastolic blood pressure (mm Hg)	77 ± 1.4	76 ± 1.5	−1.0 ± 1.22	79 ± 1.4	78 ± 1.5	−0.9 ± 1.25	0.285	0.953
Total cholesterol (mg/dL)	199 ± 6.3	190 ± 6.5	−9.4 ± 3.73	184 ± 6.5	174 ± 6.6	−10.2 ± 3.77	0.001	0.877
HDL cholesterol (mg/dL)	57 ± 2.5	50 ± 2.5	−6.4 ± 1.62	51 ± 2.5	46 ± 2.6	−5.7 ± 1.63	<0.0001	0.759
LDL cholesterol (mg/dL)	125 ± 5.0	120 ± 5.2	−4.4 ± 3.48	111 ± 5.1	110 ± 5.2	−1.2 ± 3.50	0.259	0.516
Non-HDL cholesterol (mg/dL)	143 ± 5.5	140 ± 5.7	−3.1 ± 3.36	133 ± 5.6	129 ± 5.7	−4.5 ± 3.39	0.122	0.769
Triglycerides (mg/dL)	103 ± 10.0	102 ± 10.5	−0.3 ± 7.93	123 ± 10.2	116 ± 10.5	−6.6 ± 7.94	0.542	0.575
Glucose (mg/dL)	92 ± 1.3	92 ± 1.4	0.6 ± 1.10	94 ± 1.4	91 ± 1.4	−2.9 ± 1.18	0.174	0.034
Insulin (μIU/mL)	9.6 ± 1.43	10.0 ± 1.45	0.49 ± 0.68	8.0 ± 1.46	7.4 ± 1.48	−0.58 ± 0.69	0.929	0.264
HOMA-IR	2.2 ± 0.32	2.3 ± 0.33	0.13 ± 0.16	1.8 ± 0.34	1.7 ± 0.34	−0.13 ± 0.17	0.984	0.250

Data are least squared (LS) means ± SE of the LS means.

There were no statistically significant differences between groups at baseline for any outcomes presented in this table.

Fasting blood outcomes were assessed in serum.

HOMA-IR was calculated as ((fasting glucose mg/dL × fasting insulin μIU/mL)/405).

### Lipoprotein particle numbers

The adoption of the MED diet, independent of mushroom intake, reduced buoyant HDL2b (main effect of time *P* = 0.003). No changes were found for other lipoprotein particles (Table 3). The adoption of a MED diet with mushrooms trended toward a reduction (improvement) in dense LDL III and had a medium effect size (−0.54) using Cohen’s d (Supplemental Table 3).

**TABLE 3:** Effects of consuming a MED diet with or without mushrooms for 8 wk on fasting serum lipoprotein particle numbers

Outcome	MED-Control			MED-Mushroom			*P* values	
	Baseline	Post	Change	Baseline	Post	Change	Time	Time × Group
VLDL particles (nmol/L)	62 ± 6.4	62 ± 6.9	−0.6 ± 6.43	77 ± 6.5	77 ± 6.8	0.7 ± 6.44	0.989	0.892
Total LDL particles (nmol/L)	927 ± 32.0	931 ± 33.3	4.2 ± 23.16	843 ± 32.7	834 ± 33.5	−8.9 ± 23.23	0.887	0.690
Non-HDL particles (nmol/L)	989 ± 34.5	994 ± 35.9	4.5 ± 23.88	920 ± 35.3	911 ± 36.1	−9.4 ± 23.99	0.888	0.681
Remnant lipoprotein (nmol/L)	129 ± 7.8	135 ± 8.2	6.3 ± 6.41	126 ± 8.0	136 ± 8.2	10.8 ± 6.42	0.067	0.623
Dense LDL III (nmol/L)	247 ± 20.7	270 ± 21.4	22.8 ± 13.71	259 ± 21.1	246 ± 21.6	−12.8 ± 13.79	0.612	0.069
Dense LDL IV (nmol/L)^1^	88 ± 4.2	89 ± 4.5	1.6 ± 3.86	73 ± 4.3	74 ± 4.5	0.6 ± 3.86	0.681	0.858
Total HDL particles (nmol/L)	7407 ± 130.2	7458 ± 137.6	51.5 ± 110.08	7380 ± 133.2	7144 ± 137.2	−235.9 ± 110.12	0.244	0.069
Buoyant HDL 2b (nmol/L)	2344 ± 101.3	2239 ± 105.6	−105.1 ± 73.69	2269 ± 103.6	2045 ± 106.1	−224.3 ± 73.91	0.003	0.255

Data are least squared (LS) means ± SE of the LS means.

1 There were no statistically significant differences between groups at baseline for the outcomes presented in this table, except dense LDL IV (*P* = 0.019).

### Markers of inflammation

The adoption of the MED diet, independent of mushroom intake, reduced apolipoprotein A1 and increased lipoprotein(a) concentrations. There were no changes in hs-CRP, apolipoprotein B, or homocysteine (Table 4).

**TABLE 4:** Effects of consuming a MED diet with or without mushrooms for 8 wk on fasting serum markers of inflammation

Outcome	MED-Control			MED-Mushroom			*P* values	
	Baseline	Post	Change	Baseline	Post	Change	Time	Time × Group
hs-CRP (mg/L)	1.88 ± 0.46	1.67 ± 0.48	−0.210 ± 0.313	2.39 ± 0.47	2.21 ± 0.48	−0.174 ± 0.310	0.390	0.935
Lipoprotein(a) (mg/dL)	23.1 ± 5.43	28.1 ± 5.47	4.92 ± 1.91	20.5 ± 5.56	28. 6 ± 5.60	8.06 ± 2.01	**<**0.0001	0.248
Apolipoprotein B (mg/dL)	95 ± 3.6	96 ± 3.7	1.0 ± 2.11	89 ± 3.7	90 ± 3.7	0.8 ± 2.13	0.565	0.953
Apolipoprotein A1 (mg/dL)	144 ± 4.5	130 ± 4.7	−14.3 ± 3.33	142 ± 4.6	125 ± 4.7	−16.7 ± 3.34	**<**0.0001	0.612
Homocysteine (μmol/L)	9.0 ± 0.31	8.8 ± 0.32	−0.25 ± 0.24	8.6 ± 0.31	8.7 ± 0.32	0.17 ± 0.24	0.812	0.208

Data are least square (LS) means ± SE of the LS means.

Abbreviations: hs-CRP, high-sensitivity C-reactive protein.

There were no statistically significant differences between groups at baseline for any outcomes presented in this table.

## Discussion

To the best of our knowledge, this study is the first to assess the effects of chronic fresh mushroom consumption as part of a fully controlled dietary intervention on indices of cardiometabolic health. Consistent with our hypothesis, the adoption of a MED-mushroom diet compared with the MED-control diet without mushrooms reduced (improved) fasting serum glucose with a small effect size. The adoption of a MED diet, independent of mushroom consumption, reduced total cholesterol, HDL cholesterol, buoyant HDL2b, and apolipoprotein A1, and increased lipoprotein(a). Each of these changes occurred within the ranges of clinical normalcy for these healthy participants. As such, they may be considered subclinical changes.

Results from this work are partly consistent with previous studies investigating the effects of chronic (8 wk or longer) mushroom consumption on similar traditional risk factors for CMDs. Results from one study including healthy individuals who consumed 8 ounces of fresh *A. bisporus* thrice weekly indicate reductions (improvements) in fasting blood triglycerides and glucose during the 6-mo period of purposeful weight loss, relative to baseline [31]. Another 8-wk study among individuals with human immunodeficiency virus found participants who consumed 15 g dried *P. ostreatus* daily had reduced (improved) fasting blood triglycerides, compared with baseline [32]. Two studies including individuals with type 2 diabetes mellitus indicate consumption of *P. ostreatus* reduces (improves) several cardiometabolic indices including fasting glucose, LDL- and total cholesterol, and triglycerides [33,34]. The authors of 1 of these studies also reported reductions (improvements) in blood pressure and hemoglobin A1c and an increase in HDL cholesterol, compared with baseline [34]. In contrast, individuals with prediabetes who consumed 100 g of fresh mushrooms daily for 16 wk did not have any of the aforementioned cardiometabolic health benefits [35]. Taken together, the modest and seemingly inconsistent changes in traditional CMD risk factors demonstrated in this research may be attributed to the relatively healthy study population, whereas the majority of the other studies described here included either a purposeful weight loss intervention or clinical populations.

Evidence from a year 2020 systematic review and meta-analysis indicates the consumption of a Mediterranean-style diet compared with no treatment, usual care, or advice to follow a different diet, results in greater beneficial changes in several CMD risk factors, including HDL cholesterol [36]. Inconsistent with this, we found adoption of a MED diet, independent of mushroom intake, reduced HDL cholesterol (Table 2). Though this finding is considered less favorable, mean HDL concentrations were clinically normal at all timepoints. At present, it is unclear why this response occurred. Interestingly, we have observed a reduction in HDL cholesterol concurrent with a reduction in total cholesterol in 2 other fully controlled randomized controlled trials (conducted by our group) when participants switched from consuming their usual relatively unhealthy dietary pattern to a healthy dietary pattern [[37], [38], [39]].

We are not aware of research investigating the effects of mushroom consumption on lipoprotein particle sizes. Results from this work indicate the adoption of a MED diet, independent of mushrooms, reduced (worsened) fasting buoyant HDL2b concentrations. We also found the adoption of a MED diet with mushrooms trended toward a reduction (improvement) in dense LDL III, an emerging risk factor for cardiovascular disease. Recent research indicates that small dense LDL (for example, LDL III) is the most atherogenic lipoprotein [40] because of its ability to penetrate easily into the arterial wall and may serve as an important screening tool for cardiovascular disease [41]. Notably, small, dense LDL concentrations have been shown to predict the coronary heart disease risk, even when LDL cholesterol concentrations are clinically normal [42]. Although these results are intriguing and suggest mushroom consumption may augment improvements in some cardiometabolic health indicators, they require replication and further clinical and mechanistic investigation.

The role of inflammation in the development and progression of CMDs has been established [43]. Despite literature describing an anti-inflammatory and antioxidant role of mushroom-derived bioactives (for example, L-ergothioneine, β-glucans) [44,45], few studies have reported on the effects of whole mushroom consumption on inflammatory markers. Previous experimental research indicates greater mushroom consumption reduces (improves) fasting C-reactive protein (CRP) [46] and hs-CRP [31]. Inconsistent with these reports, we found no change in hs-CRP with the adoption of either MED diet. In contrast, we observed an increase in lipoprotein(a) and a decrease in apolipoprotein A1 over time with the adoption of either MED diet. Although these findings are contrary to previous work indicating adherence to a MED diet attenuates the markers of inflammation [47], it is noteworthy that mean concentrations of all markers of vascular inflammation were within normal clinical ranges at both time points.

As briefly described in the introduction, mushrooms have been consumed for thousands of years for nutritional and medicinal purposes and are regarded as a functional food. Although narrative reviews [[6], [7], [8]] describe multiple bioactive compounds in mushrooms and potential cardiometabolic health benefits, a majority of literature describes the findings from a compound perspective in which investigators studied compounds isolated from mushrooms. For example, polysaccharides, such as beta-glucans, have demonstrated hypoglycemic properties in diabetic mice/rats [8]. Improvements in fasting/postprandial glucose and insulin are attributed to the upregulation of adiponectin and GLUT-4 genes, along with the stimulation of insulin secretion from pancreatic β-cells [44]. Lovastatin is another mushroom-derived compound that has demonstrated hypolipidemic effects in hypercholesterolemic rats [[48], [49], [50]]. Improvements in blood lipids including total, LDL, and HDL cholesterol, along with triglycerides, are attributed to the inhibition of the key enzyme in cholesterol biosynthesis, 3-Hydroxy-3-methylglutaryl–coenzyme A reductase (HMG-CoA reductase) [44]. Although these findings suggest a plausible mechanism of action for why mushrooms may promote cardiometabolic health, the findings should be interpreted with caution given most studies were conducted in cell and animal models and use isolated compounds derived from mushrooms administered at relatively higher amounts or concentrations than naturally found in whole, dietary mushrooms. It is worth noting that our recent work includes the documentation of >10,000 different compounds among 7 mushroom varieties [51], highlighting that mushrooms are a vehicle for delivering myriad nutrients/compounds that may influence health. Finally, because this study was not designed to assess a compound-specific mechanism (for example, a reductionist study), but instead to examine the influences of consuming a multicompound food, a potential mechanism of action cannot be confirmed here.

Regarding potential limitations, as described in the statistical analysis section, this study was not designed with power calculations. Instead, *n* = 30 participants/group was selected consistent with that the requirement for consideration of use by the 2015 Dietary Guidelines Advisory Committee in creating future DGA [29]. It is important that future research assessing the impacts of mushroom consumption on CMD risk factors be suitably powered. Another limitation inherent to nutrition research is the lack of blinding of participants in the mushroom group, given they were provided with fresh mushrooms weekly.

There are several strengths of this research including the use of a randomized, controlled (full-feed) study design. Although the study coordinator was responsible for providing the intervention foods to participants and completing participant testing, most study outcomes were assessed by external laboratories that were unaware of participant group assignment. All data were de-identified, double-entered by independent researchers, and cross-checked for accuracy, further safeguarding any risk of bias. Data analysts were not involved in data collection and were blinded to group assignments. The study was also designed with the use of valid and reliable outcome assessments in which study personnel were trained to collect data following standard operating procedures. All participants had high adherence to the protocol (mean dietary adherence 92%) and avoided other interventions (for example, supplements, changes in physical activity, etc.). There was a high completion rate of participants (82%) and data were analyzed using an intention-to-treat analysis.

The COVID-19 pandemic has had profound impacts on all sectors of life, including the supply chain, which consequently impacted the food availability during the time this clinical trial was conducted. In response to the unforeseen challenges posed by fluctuating food availability, the study team was required to adapt by establishing guidelines for acceptable substitutions when a protocol food was unavailable. In general, a substitution was deemed acceptable if it involved replacing a protocol food with another protocol food belonging to the same food group (for example, a vegetable for a vegetable). When possible, participants were advised to substitute a different variety or a closely related food (for example, sweet potato for a white potato; red pepper for green pepper; baby spring mix for iceberg lettuce; mixed berries for strawberries; salmon for tilapia). All deviations from the protocol were reviewed and categorized as an acceptable substitution or nonacceptable substitution/addition. This meticulous approach ensured that the study’s scientific rigor and objectives remained intact despite the challenges presented by external factors beyond our control.

Given the modest and mainly neutral responses in CMD risk factors observed with this population of healthy middle-aged and older adults, future work may consider examining the effects of consuming mushrooms on these health-related outcomes in populations that may confer greater benefits (for example, those with hypertension, dyslipidemia, prediabetes, etc.) while they consume different dietary patterns, including more typical self-chosen, less healthy Western patterns.

In conclusion, the consumption of a Mediterranean-style healthy dietary pattern with 1 serving per day of whole *Agaricus bisporus* and *Pleurotus ostreatus* mushrooms for 2 mo improved fasting serum glucose, but not any other established or emerging CMD risk factors among middle-aged and older adults classified as overweight or class I obese but with clinically normal CMD risk factors.

## Author contributions

The authors’ responsibilities were as follows – CNU, MRO, NAR, MT, NFK, WWC: designed the research; CNU, JNS: conducted the research; CNU, REC, ASH, WWC: analyzed data; CNU: wrote the article; all authors: provided editorial assistance during manuscript development; CNU, WWC: are responsible for the final content; and all authors: read and approved the final manuscript.

## Conflict of interest

The authors declare no conflicts of interest.

## Funding

This research was funded by the Mushroom Council. The funders had no role in the design of the study; in the collection, analyses, or interpretation of data; in the writing of the manuscript; or in the decision to publish the results.

## Data availability

Data described in the manuscript are available upon request.
