# Supplementary material for: Consuming Mushrooms When Adopting a Healthy Mediterranean-Style Dietary Pattern Does Not Influence Short-Term Changes of Most Cardiometabolic Disease Risk Factors in Healthy Middle-Aged and Older Adults
Source: J Nutr. 2023 Dec 20;154(2):574–82. doi: 10.1016/j.tjnut.2023.12.026 (PMC10997904; doi:10.1016/j.tjnut.2023.12.026)
Supplement: Multimedia component 1 [file mmc1.docx]

**Supplemental Table 1.** Servings of Food Groups in the USDA Healthy Mediterranean-Style Dietary Pattern and Study Diets

| Dietary Pattern Calorie Level | 2,000 | | 2,400 | | 2,800 | |
| --- | --- | --- | --- | --- | --- | --- |
| Food Group | USDA  Med-HDP^a^ | Study  Med-HDP^b^ | USDA  Med-HDP | Study  Med-HDP | USDA  Med-HDP | Study  Med-HDP |
| Vegetables (total), c-eq/day | 2.5^c^ | 2.5 | 3 | 3 | 3.5 | 3.5 |
| Dark-green (c-eq/wk) | 1.5 | 3 | 2 | 3.5 | 2.5 | 4 |
| Red and orange (c-eq/wk) | 5.5 | 5.5 | 6 | 6.5 | 7 | 7.5 |
| Legumes (beans and peas) (c-eq/wk) | 1.5 | 1.5 | 2 | 2 | 2.5 | 2.5 |
| Starchy vegetables (c-eq/wk) | 5 | 5 | 6 | 5 | 7 | 7.5 |
| Other vegetables (c-eq/wk) | 4 | 3 | 5 | 4 | 5.5 | 4 |
| Fruits (total), c-eq/day | 2.5 | 2 | 2.5 | 2 | 3 | 2.5 |
| Grains (total), oz-eq/day | 6 | 6 | 8 | 7.5 | 10 | 9.5 |
| Whole grains (oz-eq/day) | 3 | 3.5 | 4 | 4.5 | 5 | 5 |
| Refined grains (oz-eq/day) | 3 | 2.5 | 4 | 3.5 | 5 | 4 |
| Dairy, c-eq/day | 2 | 2 | 2.5 | 2 | 2.5 | 2.5 |
| Protein foods, oz-eq/day | 6.5 | 6.5 | 7.5 | 7.5 | 8 | 8.5 |
| Seafood (oz-eq/wk) | 15 | 15 | 16 | 16 | 17 | 17 |
| Meats, poultry, eggs (oz-eq/wk) | 26 | 26 | 31 | 32.5 | 33 | 34.5 |
| Nuts, seeds, soy (oz-eq/wk) | 5 | 4 | 5 | 5 | 6 | 6.5 |
| Oils, g/day | 27 | 21.5 | 31 | 24 | 36 | 31.5 |
| Limit on calories for other uses | 260 | 116 | 300 | 113 | 350 | 117 |

^a^USDA healthy Mediterranean-style eating pattern recommended amounts of food from each food group following Appendix 4 of the 2015-2020 Dietary Guidelines for Americans.

^b^Average daily or weekly amounts of foods from each food group/subgroup in the control dietary pattern. Participants in the mushroom group consumed more total vegetables (84 g/d or ~1 c-eq/day) not reflected here. Values listed for the study Med-HDP are rounded to the nearest half number.

^c^Daily amount of food from each group is listed. Subgroup amounts for vegetable and protein foods are per week.

Abbreviations: c-eq/day: cup-equivalence per day; c-eq/wk: cup-equivalence per week; g/day: grams/day; Med-HDP: Mediterranean-Style Healthy Dietary Pattern; oz-eq/day: ounce-equivalence per day; USDA: United States Department of Agriculture

**Supplemental Figure 1.** Total HEI score for baseline dietary intake data and the dietary

intervention following a USDA Healthy Mediterranean-Style Dietary Pattern

HEI scores were calculated using all available baseline dietary intake data from ASA-24 (n=69).

The maximum possible HEI score is 100.

U.S. Average HEI score for Americans age 2+ years is 58.

**Supplemental Table 2.** Fasted serum clinical markers and plasma L-ergothioneine

|  | MED-Control | | | MED-Mushroom | | | P-values | |
| --- | --- | --- | --- | --- | --- | --- | --- | --- |
| Outcome | Baseline | Post | Change | Baseline | Post | Change | Time | Time x Group |
| BUN (mg/dL) | 14 ± 0.6 | 16 ± 0.6 | 2.0 ± 0.61 | 13 ± 0.6 | 16 ± 0.6 | 2.4 ± 0.65 | <.0001 | 0.724 |
| Creatinine (mg/dL) | 0.86 ± 0.02 | 0.88 ± 0.02 | 0.018 ± 0.013 | 0.84 ± 0.02 | 0.89 ± 0.02 | 0.050 ± 0.014 | 0.001 | 0.087 |
| BUN:Creatinine | 16 ± 0.8 | 18 ± 0.9 | 2.0 ± 0.76 | 16 ± 0.8 | 18 ± 0.9 | 1.8 ± 0.82 | 0.001 | 0.884 |
| eGFR (mL/min/1.73 m^2^) | 95 ± 1.9 | 93 ± 2.0 | -1.6 ± 1.34 | 97 ± 2.0 | 92 ± 2.0 | -4.6 ± 1.44 | 0.003 | 0.132 |
| ALT (U/L) | 22 ± 1.5 | 23 ± 1.6 | 0.4 ± 1.19 | 21 ± 1.6 | 21 ± 1.6 | 0.03 ± 1.27 | 0.791 | 0.817 |
| AST (U/L) | 24 ± 1.0 | 22 ± 1.1 | -1.7 ± 1.08 | 22 ± 1.1 | 21 ± 1.1 | -0.7 ± 1.15 | 0.146 | 0.528 |
| L-ergothioneine (uM) | 1.2 ± 0.19 | 1.1 ± 0.21 | -0.10 ± 0.25 | 1.1 ± 0.19 | 4.8 ± 0.21 | 3.64 ± 0.26 | <.0001 | <.0001 |

Data are least squared (LS) means $\pm$ SE of the LS means

There were no statistically significant differences between groups at baseline for any outcomes presented in this table.

Fasting blood outcomes were assessed in serum, except for L-ergothioneine which was measured in plasma.

Abbreviations: ALT: Alanine transaminase; AST: aspartate aminotransferase; BUN: blood urea nitrogen; eGFR: estimated glomerular filtration rate

**Supplemental Table 3. Post-intervention LS means and differences between means**

| Outcome | MED-Control  LS Mean  Post | MED-Mushroom LS Mean  Post | Difference between means | P-value |
| --- | --- | --- | --- | --- |
| Systolic blood pressure (mm Hg) | 114 | 115 | -0.3 | 0.866 |
| Diastolic blood pressure (mm Hg) | 77 | 77 | -0.8 | 0.487 |
| Total cholesterol (mg/dL) | 182 | 181 | 1.6 | 0.703 |
| HDL cholesterol (mg/dL) | 49 | 49 | 0.2 | 0.882 |
| LDL cholesterol (mg/dL) | 114 | 114 | 0.1 | 0.983 |
| Non-HDL cholesterol (mg/dL) | 133 | 131 | 1.9 | 0.620 |
| Triglycerides (mg/dL) | 104 | 105 | -1.0 | 0.909 |
| Glucose (mg/dL) | 92 | 90 | 2.1 | 0.082 |
| Insulin (μIU/mL) | 9.5 | 8.5 | 1.01 | 0.243 |
| HOMA-IR | 2.2 | 2.0 | 0.17 | 0.427 |
| VLDL particles (nmol/L) | 63 | 69 | -6.2 | 0.350 |
| Total LDL particles (nmol/L) | 894 | 865 | 29.2 | 0.261 |
| Non-HDL particles (nmol/L) | 957 | 934 | 23.2 | 0.374 |
| Remnant lipoprotein (nmol/L) | 133 | 136 | -2.5 | 0.714 |
| Dense LDL III (nmol/L) | 264 | 235 | 29.4 | 0.074 |
| Dense LDL IV (nmol/L) | 85 | 80 | 5.1 | 0.260 |
| Total HDL particles (nmol/L) | 7467 | 7219 | 247.6 | 0.047 |
| Buoyant HDL 2b (nmol/L) | 2221 | 2155 | 66.9 | 0.303 |
| hs-CRP (mg/L) | 2.09 | 2.11 | -0.020 | 0.955 |
| Lipoprotein(a) (mg/dL) | 28.1 | 31.1 | -3.04 | 0.072 |
| Apolipoprotein B (mg/dL) | 92 | 91 | 0.6 | 0.802 |
| Apolipoprotein A1 (mg/dL) | 129 | 128 | 1.3 | 0.658 |
| Homocysteine (μmol/L) | 8.5 | 8.8 | -0.22 | 0.365 |
| BUN (mg/dL) | 16 | 16 | 0.0 | 0.968 |
| Creatinine (mg/dL) | 0.87 | 0.89 | -0.017 | 0.228 |
| BUN:Creatinine | 18 | 18 | 0.3 | 0.734 |
| eGFR (mL/min/1.73 m2) | 94 | 92 | 1.8 | 0.259 |
| ALT (U/L) | 21 | 21 | 0.3 | 0.867 |
| AST (U/L) | 22 | 21 | 0.5 | 0.565 |
| L-ergothioneine (uM) | 1.4 | 4.5 | -3.11 | <.0001 |

*Data were analyzed using a linear model using the PROC GLM command in SAS version 9.4 to predict post-intervention values and differences between groups at post-intervention. Covariates in this model included age, sex, BMI, and baseline values.

Abbreviations: ALT: Alanine transaminase; AST: aspartate aminotransferase; BUN: blood urea nitrogen; eGFR: estimated glomerular filtration rate; HDL: high-density lipoprotein; HOMA IR: homeostatic model assessment for insulin resistance; hs-CRP: high-sensitivity C-reactive protein; LDL: low-density lipoprotein; VLDL: very-low-density lipoprotein

**Supplemental Table 4.** Unadjusted means, SD, sample size, and Cohen’s d effect size by outcome

|  | MED-Control | | | | | | | | | MED-Mushroom | | | | | | | | | Cohen's d^1^ |
| --- | --- | --- | --- | --- | --- | --- | --- | --- | --- | --- | --- | --- | --- | --- | --- | --- | --- | --- | --- |
|  | Baseline | | | Post | | | Change | | | Baseline | | | Post | | | Change | | |  |
| Outcome | Mean | SD | n | Mean | SD | n | Mean | SD | n | Mean | SD | n | Mean | SD | n | Mean | SD | n |  |
| Systolic blood pressure  (mm Hg) | 115 | 10.2 | 38 | 113 | 10.5 | 30 | -1.7 | 8.79 | 30 | 118 | 13.1 | 34 | 116 | 12.5 | 30 | -2.7 | 8.53 | 30 | -0.12 |
| Diastolic blood pressure  (mm Hg) | 77 | 6.7 | 38 | 76 | 6.3 | 30 | -1.0 | 5.66 | 30 | 79 | 11.2 | 34 | 78 | 8.0 | 30 | -1.3 | 7.83 | 30 | -0.04 |
| Total cholesterol (mg/dL) | 199 | 36.3 | 36 | 186 | 41.2 | 29 | -9.4 | 20.74 | 28 | 186 | 39.8 | 33 | 177 | 34.7 | 30 | -12.3 | 18.67 | 30 | -0.15 |
| HDL cholesterol (mg/dL) | 57 | 20.4 | 36 | 50 | 14.9 | 29 | -5.9 | 8.53 | 28 | 52 | 14.1 | 33 | 47 | 10.9 | 30 | -5.0 | 8.67 | 30 | 0.10 |
| LDL cholesterol (mg/dL) | 123 | 29.5 | 36 | 117 | 30.9 | 29 | -4.9 | 18.51 | 28 | 113 | 32.1 | 33 | 112 | 30.1 | 30 | -4.0 | 18.04 | 30 | 0.05 |
| Non-HDL cholesterol (mg/dL) | 142 | 32.2 | 36 | 136 | 35.5 | 29 | -3.6 | 19.95 | 28 | 134 | 35.2 | 33 | 130 | 32.6 | 30 | -7.3 | 15.05 | 30 | -0.21 |
| Triglycerides (mg/dL) | 103 | 57.2 | 36 | 102 | 55.4 | 29 | -1.4 | 36.67 | 28 | 123 | 61.7 | 33 | 114 | 68.9 | 30 | -10.9 | 48.29 | 30 | -0.22 |
| Glucose (mg/dL) | 91 | 8.6 | 37 | 92 | 8.9 | 30 | 0.1 | 5.47 | 30 | 94 | 8.3 | 31 | 91 | 6.6 | 29 | -2.9 | 6.79 | 27 | -0.49 |
| Insulin (μIU/mL) | 10.5 | 10.10 | 36 | 11.4 | 12.69 | 29 | 0.23 | 4.54 | 28 | 8.3 | 5.54 | 33 | 7.2 | 4.54 | 30 | -1.21 | 2.66 | 30 | -0.39 |
| HOMA-IR | 2.4 | 2.30 | 36 | 2.6 | 2.83 | 29 | 0.07 | 1.02 | 28 | 1.8 | 1.03 | 30 | 1.6 | 1.03 | 29 | -0.27 | 0.65 | 27 | -0.39 |
| VLDL particles (nmol/L) | 62 | 38.6 | 36 | 61 | 37.9 | 29 | -0.8 | 27.75 | 28 | 77 | 36.8 | 33 | 75 | 41.9 | 30 | -2.4 | 40.94 | 30 | -0.05 |
| Total LDL particles (nmol/L) | 929 | 192.1 | 36 | 913 | 193.7 | 29 | 4.5 | 135.56 | 28 | 852 | 215.2 | 33 | 848 | 176.3 | 30 | -29.7 | 108.33 | 30 | -0.28 |
| Non-HDL particles (nmol/L) | 992 | 212.9 | 36 | 974 | 206.1 | 29 | 3.7 | 151.08 | 28 | 930 | 227.7 | 33 | 923 | 196.5 | 30 | -32.0 | 95.81 | 30 | -0.28 |
| Remnant lipoprotein (nmol/L) | 128 | 52.2 | 36 | 132 | 49.7 | 29 | 6.4 | 31.06 | 28 | 127 | 44.7 | 33 | 136 | 35.7 | 30 | 7.6 | 36.94 | 30 | 0.03 |
| Dense LDL III (nmol/L) | 238 | 107.9 | 36 | 267 | 141.2 | 29 | 18.5 | 77.03 | 28 | 262 | 132.8 | 33 | 250 | 129.5 | 30 | -21.4 | 70.99 | 30 | -0.54 |
| Dense LDL IV (nmol/L) | 90 | 29.5 | 36 | 91 | 33.3 | 29 | 2.1 | 23.58 | 28 | 74 | 21.0 | 33 | 76 | 17.5 | 30 | -0.4 | 18.02 | 30 | -0.12 |
| Total HDL particles (nmol/L) | 7473 | 910.8 | 36 | 7425 | 802.2 | 29 | 100.6 | 646.90 | 28 | 7393 | 681.0 | 33 | 7176 | 707.7 | 30 | -237.4 | 532.82 | 30 | -0.57 |
| Buoyant HDL 2b (nmol/L) | 2431 | 865.9 | 36 | 2257 | 645.3 | 29 | -74.5 | 403.78 | 28 | 2278 | 611.3 | 33 | 2113 | 522.1 | 30 | -212.0 | 382.73 | 30 | -0.35 |
| hs-CRP (mg/L) | 2.34 | 2.87 | 36 | 2.03 | 2.70 | 28 | -0.221 | 0.709 | 27 | 2.39 | 3.14 | 33 | 2.12 | 3.06 | 30 | -0.361 | 2.127 | 30 | -0.09 |
| Lipoprotein(a) (mg/dL) | 24.4 | 29.41 | 36 | 27.7 | 31.76 | 29 | 5.00 | 9.86 | 28 | 20.9 | 28.43 | 33 | 29.7 | 37.70 | 30 | 7.98 | 10.16 | 30 | 0.30 |
| Apolipoprotein B (mg/dL) | 94 | 20.0 | 36 | 94 | 22.5 | 29 | 0.6 | 11.87 | 28 | 90 | 23.5 | 33 | 91 | 22.6 | 30 | -1.2 | 10.27 | 30 | -0.16 |
| Apolipoprotein A1 (mg/dL) | 145 | 35.9 | 36 | 129 | 24.9 | 29 | -13.1 | 18.72 | 28 | 142 | 25.3 | 33 | 128 | 20.4 | 30 | -15.4 | 16.86 | 30 | -0.13 |
| Homocysteine (μmol/L) | 8.7 | 2.35 | 36 | 8.3 | 1.73 | 29 | -0.24 | 1.16 | 28 | 8.6 | 2.02 | 33 | 8.7 | 2.09 | 30 | 0.14 | 1.32 | 30 | 0.31 |
| BUN (mg/dL) | 13 | 4.1 | 37 | 15 | 2.9 | 30 | 2.3 | 3.57 | 30 | 14 | 3.2 | 31 | 16 | 4.0 | 29 | 2.5 | 3.27 | 27 | 0.07 |
| Creatinine (mg/dL) | 0.84 | 0.15 | 37 | 0.84 | 0.12 | 30 | 0.022 | 0.067 | 30 | 0.83 | 0.10 | 31 | 0.88 | 0.14 | 29 | 0.051 | 0.072 | 27 | 0.43 |
| BUN:Creatinine | 16 | 6.1 | 37 | 18 | 4.3 | 30 | 2.0 | 4.82 | 30 | 16 | 4.0 | 31 | 18 | 4.2 | 29 | 1.9 | 3.55 | 27 | -0.03 |
| eGFR (mL/min/1.73 m2) | 95 | 14.5 | 37 | 95 | 14.2 | 30 | -1.8 | 6.66 | 30 | 96 | 13.9 | 31 | 92 | 15.1 | 29 | -4.6 | 7.95 | 27 | -0.38 |
| ALT (U/L) | 21 | 11.6 | 37 | 22 | 11.5 | 30 | -0.1 | 7.84 | 30 | 20 | 7.9 | 31 | 20 | 8.9 | 29 | -1.0 | 4.60 | 27 | -0.14 |
| AST (U/L) | 23 | 8.4 | 37 | 21 | 5.3 | 30 | -1.9 | 5.81 | 30 | 22 | 6.1 | 31 | 21 | 5.0 | 29 | -1.0 | 6.20 | 27 | 0.15 |
| L-ergothioneine (uM) | 1 | 0.6 | 37 | 1 | 0.5 | 30 | -0.1 | 0.32 | 30 | 1 | 0.4 | 34 | 5 | 2.1 | 30 | 3.7 | 2.09 | 30 | 2.51 |

^1^Cohen’s d effect size is estimated using the mean change values (mushroom-control)

Abbreviations: ALT: Alanine transaminase; AST: aspartate aminotransferase; BUN: blood urea nitrogen; eGFR: estimated glomerular filtration rate; HDL: high-density lipoprotein; HOMA IR: homeostatic model assessment for insulin resistance; hs-CRP: high-sensitivity C-reactive protein; LDL: low-density lipoprotein; VLDL: very-low-density lipoprotein
